# Rupture of Non-communicating Rudimentary Horn Pregnancy at 15 Weeks with Previous Normal Pregnancies: A Case Report

**DOI:** 10.31729/jnma.5104

**Published:** 2020-08-31

**Authors:** Sanyukta Rajbhandary, Anamika Das, Mausam Rai, Archana Kumari Sah

**Affiliations:** 1 Department of Obstetrics and Gynecology, B.P Koirala Institute of Health Sciences, Ghopa, Dharan, Nepal

**Keywords:** *acute abdomen*, *case report*, *hemoperitoneum*, *mullerian anomalies*, *rudimentary horn*

## Abstract

Rudimentary horn is a mullerian anomaly that is a variant of unicornuate uterus. Rudimentary horn pregnancies are rare and associated with increased maternal morbidity and mortality. Diagnosis of rudimentary horn pregnancy and its rupture in a woman with previous vaginal delivery is challenging. Although ultrasonography is an important diagnostic tool, it has low sensitivity in making diagnosis of ruptured rudimentary horn pregnancy. Therefore, clinicians should have high index of suspicion in such cases. We report a case of G3P2L2 at 15 weeks period of gestation referred to our centre as a case of intrauterine pregnancy with acute abdomen. She underwent emergency laparotomy and was found to have ruptured rudimentary horn intraoperatively. Excision of the ruptured rudimentary horn and ipsilateral salpingectomy was done and the patient had an uneventful postoperative recovery.

## INTRODUCTION

The unicornuate uterus is a result of abnormal or failed development of one of the paired mullerian ducts.^1^ A unicornuate uterus can be present alone or with a rudimentary horn or bulb on the opposite side. Rupture through the wall of the vascular rudimentary horn is related to sudden and severe intra peritoneal hemorrhage and shock.^2^ About 72-85% of rudimentary horn are non-communicating with the cavity.^3^ Incidence of rudimentary horn pregnancies is estimated at 1:76,000-1:160,000 pregnancies.^4^ The first case of uterine rupture associated with rudimentary horn pregnancy was reported in 1669 by Mauriceau and Vassal.^5^

## CASE REPORT

A 25 year old G3P2L2 at 15 weeks of pregnancy was referred to the emergency of our hospital from another Centre with diagnosis of pregnancy with acute abdomen. She had previous two uneventful vaginal deliveries at home with last child birth 3 years back. She presented to our emergency with pain abdomen for 2 days and vomiting for 1 day. There was no complaint of per vaginal bleeding. She gave no history of medical abortion or any instrumentation done. She had gone to a rural hospital for this complaint where she was conservatively managed and an obstetric ultrasound was done documenting a 15 weeks single live intrauterine pregnancy. But when her condition started deteriorating, she was referred to our centre for further management. Our hospital, B.P. Koirala Institute of Health Sciences is a tertiary care referral hospital in Eastern Nepal.

On examination in our Obstetric emergency, she was ill looking with severe pallor. Her pulse was 140 beats/min, blood pressure 90/50 mmhg, respiratory rate 28 cycles/min. On abdominal examination, there was generalized tenderness. Bowel sound was present. On per speculum examination of vagina, there was no cervical pathology and no active bleeding was noted. On per vaginal examination, the uterine size could not be assessed due to tenderness and voluntary guarding. There was fullness in anterior fornix and tenderness in bilateral fornices.

**Figure 1.**
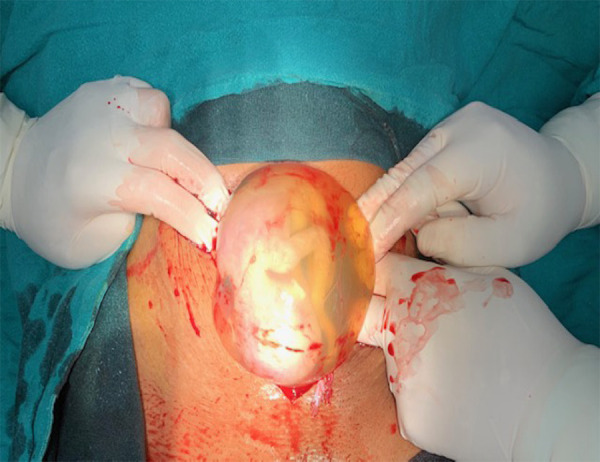
Fetus with intact amniotic sac in the right ruptured rudimentary horn of unicornuate uterus.

**Figure 2.**
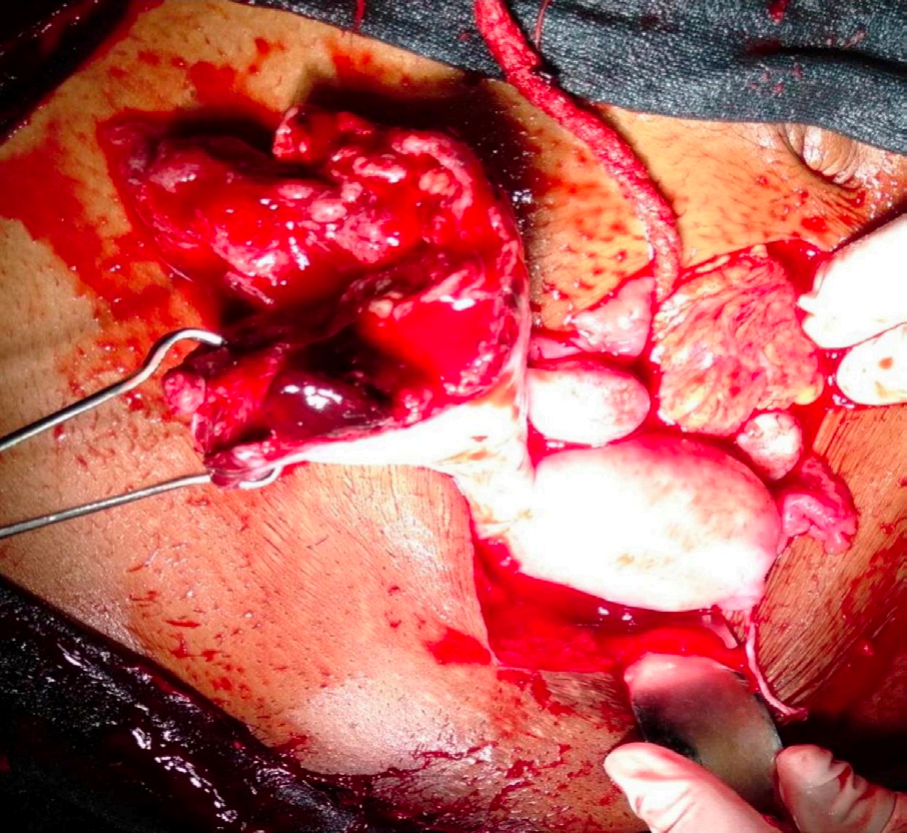
Ruptured right rudimentary horn of unicornuate uterus.

After admission, investigations were sent, blood arranged and resuscitation started immediately. Investigations sent in our hospital were: B positive blood group, Hb: 2.8 gm/dl, RBS: 78mg/dL, HIV, HepBsAg and VDRL negative. Ultrasonography done in emergency showed intrauterine pregnancy of 15 weeks period of gestation with no cardiac activity and hemoperitoneum. The patient was immediately prepared for emergency laparotomy. Abdomen was opened by midline infraumbilical incision. There was hemoperitoneum of approximately 1500ml. On further exploration, there was a fetus lying within the intact amniotic sac (Fig 1) in the ruptured right rudimentary horn of the unicornuate uterus. There was complete rupture of right rudimentary horn and was connected to the unicornuate uterus by a fibromuscular band (Fig 2). No intracavitary connection was noted between the unicornuate uterus and the rudimentary horn. The left ovary and fallopian were attached normally to the unicornuate uterus. Excision of the ruptured right rudimentary horn and right fallopian tube with conservation of the right ovary was done. Hemostasis was achieved and drain was kept. She was shifted to the maternal intensive care unit where she received total 8 units of blood transfusion. Her post-operative period was uneventful and she was discharged on the fifth postoperative day in a stable and satisfactory condition.

## DISCUSSION

The overall incidence of mullerian anomalies has been reported to be 3.2% in women with normal reproductive outcomes, 5% to 10% in women with recurrent first trimester miscarriages, and greater than 25% in women with late first and early second-trimester miscarriages. The unicornuate uterus constitutes approximately 20% of mullerian duct anomalies, and most patients are asymptomatic until menarche or if and when they become pregnant. In 1979, Buttram and Gibbons developed a classification system of mullerian duct anomalies based on the degree of failure of normal development. It was updated in 1988 by the American Society of Reproductive Medicine.^6^

The unicornuate uterus can be further subdivided into 4 variants according to the American Fertility Society.^6^

A1a: Unicornuate uterus with communicating cavitary rudimentary horn (10%)

A1b: Unicornuate uterus with non communicating cavitary horn (22%)

A2: Unicornuate uterus with non cavitary rudimentary horn (33%)

B: Isolated unicornuate uterus (35%)

Pregnancy in a non-communicating rudimentary horn occurs through the transperitoneal migration of the spermatozoon or transperitoneal migration of fertilized ovum.^7^ The most significant threat of rudimentary horn pregnancy is the risk of rupture because of poorly developed uterine musculature. In a review of 588 rudimentary horn pregnancies by Nahum, 13% occurred in the first trimester, 67% occurred in the second trimester and 20% occurred in the third trimester.^8^

Maternal mortality due to rupture of rudimentary horn in the 19^th^ century was 88%. The last maternal death was reported in 1960 by Linders-Kupka of Germany. The most recent estimates of maternal mortality are less than 0.5%, despite rupture rates of 50%^9^ since rupture of the rudimentary horn pregnancy is associated with increased maternal morbidity and mortality; it is of utmost importance to make early diagnosis and manage accordingly. There is low preclinical and preoperative detection (14% overall) for rudimentary horn presentations.^8^ Therefore, there should be high index of suspicion. As in our case, the patient first went to rural health care Centre, where she was being treated as a case of pregnancy with acute abdomen and referred to our centre only when her condition deteriorated.

Ultrasound is useful diagnostic tool with a sensitivity of only 26% and sensitivity decreases as pregnancy advances.^8^ Tubal pregnancy, cornual pregnancy, intrauterine pregnancy and abdominal pregnancy are common sonographic misdiagnosis. Tsafir et al reported two cases of rudimentary horn pregnancy found in first trimester and confirmed by MRI. They outlined a set of criteria for diagnosing pregnancy in the rudimentary horn. They are (i) pseudopattern of bicornuate uterus, (ii) absent visual continuity of tissue, the gestation sac and the uterine cervix; (iii) presence of myometrial tissue surrounding gestational sac.^10^ Therefore, availability of tools like ultrasound and MRI are valuable in making an accurate diagnosis. But they may not be always readily available in developing countries where resources are limited. As mentioned in our case, although ultrasound was done twice but was found to be inconclusive. So, it is very important for clinicians to have high index of suspicion and keep rupture of rudimentary horn pregnancy as a differential diagnosis when encountered in such cases.

Immediate surgery is recommended by most after diagnosis even in unruptured cases.^8^ Excision of the rudimentary horn and ipsilateral salpingectomy (to prevent a further ectopic tubal gestation), with the intention of conserving the ovary, is the recommended surgical procedure for patients desiring to maintain their fertility potential. As rudimentary horn pregnancies are associated with increased morbidity and mortality, if an asymmetric uterine horn is recognized in a non-gravid patient, elective surgical removal should be performed.^9^ Some rudimentary horn pregnancies have been successfully removed laproscopically.^11^

A case of pregnancy progressing to the third trimester (34 weeks) and resulting in live birth after a cesarean section has also been reported in korea.^12^ Rupture of rudimentary horn pregnancy is a rare entity. As preoperative and prerupture diagnosis rates are low, early diagnosis is the key to successful management. Therefore, there should be high index of suspicion and it should always be considered as a differential diagnosis in a pregnant woman presenting with acute abdomen and shock.
